# Inclusive legal education as an upstream intervention: building legal governance and capacity for health equity

**DOI:** 10.3389/fpubh.2026.1748388

**Published:** 2026-04-13

**Authors:** Fei Qi, Bin Yu, Zhoumiqi Yuan, Jing Wang, Yuan Wang

**Affiliations:** 1 School of Law, Hainan University, Haikou, China; 2 University President’s Office, Hainan University, Haikou, China; 3 The Affiliated Cancer Hospital of Zhengzhou University, Henan Cancer Hospital, Zhengzhou, China; 4 National Prosecutors College of P. R. C, Beijing, China

**Keywords:** capacity building, disability representation, empowerment, health equity, health governance, legal education inclusivity, social determinants of health, social participation

## Abstract

Achieving health equity requires attention not only to healthcare access and the social determinants of health, but also to the governance capacity of institutions that shape health-related policies and legal frameworks. This study explores inclusive legal education as a potential upstream intervention for building health governance capacity, focusing on how barriers in legal professional training may indirectly connect to the health equity agenda. Through a secondary qualitative analysis of publicly available narrative texts and official documents concerning 14 Chinese legal professionals with disabilities, this study identified three interconnected dimensions. First, these public narratives reveal that institutional environments reflecting tendencies that disability scholars characterize as ableism generate discernible barriers in legal education and professional entry pathways, barriers that could intersect with broader patterns of socioeconomic stratification recognized as social determinants of health outcomes. Second, while informal social capital serves compensatory functions when formal support systems remain underdeveloped, this reliance may inadvertently create filtering mechanisms that narrow the range of perspectives available for health policy formulation. Third, public narratives indicate that some individuals exhibit transformative agency, leveraging their legal professional identities to advocate for institutional improvement in ways that may be understood as a form of empowered social participation in health governance. These findings advance a conceptual argument: promoting inclusivity in legal education may constitute upstream capacity-building for health equity. By expanding professional diversity within legal systems, such reform may contribute to governance institutions’ capacity to develop more inclusive health policies and anti-discrimination frameworks. This research offers a conceptual framework and preliminary theoretical grounding for understanding how capacity building, social participation, and empowerment in legal education reform may conceptually connect to the broader health equity agenda.

## 1 Introduction

Health equity—defined as the absence of systematic disparities in health status and healthcare access across social groups—has emerged as a central concern in global public health discourse (1). While substantial attention has focused on healthcare service delivery and addressing immediate social determinants of health, the role of governance capacity and institutional representation in shaping health-related policies remains less fully examined (2–4). This oversight is particularly significant given that legal and policy frameworks constitute critical structural determinants that shape population health outcomes through mechanisms including anti-discrimination legislation, accessibility standards in healthcare settings, and the design of health-related social protection systems (5, 6).

Within this broader context, the composition and diversity of legal professional communities may represent an underexplored upstream factor influencing health equity. Legal professionals—as legislators, judges, prosecutors, and policy advisors—play pivotal roles in developing and interpreting health-related legal frameworks (7). When these professional communities systematically underrepresent certain populations, particularly persons with disabilities, the resulting governance structures may lack critical experiential perspectives necessary for formulating truly inclusive health policies (8). This study therefore examines inclusive legal education through a public health lens: institutional barriers that restrict entry into the legal profession narrow the compositional diversity of those who shape health-related policy, and addressing such barriers may thus constitute a form of upstream capacity building for health equity. By investigating how persons with disabilities navigate barriers in legal professional training within China’s context, this research explores the potential connections between educational inclusivity, professional diversity, and health governance capacity—thereby contributing to understanding the intersection of capacity building, social participation, and empowerment in advancing health equity.

The relevance of this connection between legal education inclusivity and health governance may be better understood by examining the mission of legal education itself. As the institutional carrier for cultivating legal professionals, legal education systems shoulder a dual mission: developing technical competencies and shaping practitioners’ understanding of justice and ethical consciousness (9). As a discipline fundamentally grounded in advocating justice and equality, legal education possesses unique advantages and is critical in advancing social inclusion and promoting educational equity (10). Fulfilling this mission necessarily requires legal education to address contemporary issues of social inclusivity, with the level of inclusion extended to persons with disabilities serving as a crucial benchmark for evaluating this commitment (11). However, reality demonstrates that this professional field dedicated to pursuing fairness and justice still faces challenges from attitudes reflective of ableism in talent cultivation and professional entry processes (12, 13), indicating substantial room for improvement in disability inclusion. More concerningly, this phenomenon is often obscured by inspirational narratives celebrating individual persons with disabilities who “overcome adversity to achieve excellence.” (14) Such narratives tend to reframe systemic institutional issues requiring structural attention into expectations for individual adaptive capacity. On the surface, this reflects an incomplete alignment between educational ideals and practical implementation in legal education; at a deeper level, it reveals ongoing tensions between traditional legal education models and educational objectives centered on cultivating ideals of equality and justice.

These theoretical concerns regarding the intersection of legal education inclusivity and health governance require empirical examination within specific national contexts. China provides a particularly instructive case for investigating these dynamics. China’s substantial population of persons with disabilities (exceeding 85 million) (15), combined with its considerable legal education system (over 600 law schools) (16), constitutes a research sample of significant representativeness for understanding how institutional barriers in legal professional training may influence the diversity of perspectives available in health-related policy development. In recent years, China has achieved notable progress in legal frameworks for disability protection, not only actively participating in the formulation of and responding to the United Nations Convention on the Rights of Persons with Disabilities (CRPD), but also establishing a domestic protection system centered on the Law on the Protection of Persons with Disabilities and the Law on Barrier-Free Environment Construction (17). These top-level designs have established principled guarantees for the equal educational rights of persons with disabilities and laid institutional foundations for protection (18). In practice, these institutional advances have enabled cohorts of students with disabilities to enter law schools and gradually develop into legal professionals successfully.

However, as supporting institutional mechanisms continue to evolve, persons with disabilities who actually enter and complete legal education often rely heavily on extraordinary personal effort and diverse social and familial support (19). This gap between macro-level rights commitments and micro-level practice has generated a critical blind spot in understanding: due to China’s lack of systematic tracking and data collection on Legal Professionals with Disabilities (LPwD), we cannot comprehensively understand what institutional barriers this population confronts, nor what “additional adaptive, demonstrative, and negotiative efforts” (20) they must undertake in order to realize their rightful educational and career development opportunities fully.

This study conceptualizes the sustained, proactive efforts persons with disabilities make to access educational resources and professional opportunities as “Strategic Negotiation.” This concept draws on two bodies of literature whose intersection this study seeks to explore in the context of legal professional training. From disability studies, it takes up work on identity management and impression work in ableist institutional environments (20, 21) and scholarship on the unacknowledged labor that disabled individuals absorb in order to participate on ostensibly equal terms (22). From social capital theory, it draws on the bonding/bridging distinction (23) and network-based accounts of how individuals positioned at the margins of professional fields mobilize relational ties to gain access (24). Strategic negotiation is proposed as a concept that brings these two dimensions together, treating identity management and capital mobilization as interdependent rather than separate processes. It emphasizes that persons with disabilities are not passive recipients of rights but agentic subjects who must actively identify institutional gaps, mobilize social capital, manage identity presentation, and repeatedly negotiate with relevant authorities, educators, and peers in daily practice. Strategic negotiation encompasses both formal reasonable accommodation application processes and informal everyday interactions; it involves individuals’ assessment and articulation of their needs and decisions about making appropriate choices in different contexts. However, due to data gaps, we cannot systematically identify the specific content of these strategic negotiations, their implementation effectiveness, or the additional energy and time costs LPwD invest in this process.

In stark contrast to this pervasive “invisibility,” a small number of successful LPwD cases have garnered significant social attention through media coverage (25). These narrative texts often present a similar pattern: attributing success primarily to individual tenacity and extraordinary perseverance, constructing them as exemplars of “triumph over disability through indomitable will.” However, this exceptionism narrative framework centered on individuals, while ostensibly highlighting the agency of persons with disabilities, may generate certain cognitive biases: (26) on one hand, it tends to reframe systemic institutional issues requiring structural attention into challenges individuals must adapt to, thereby diminishing focus on institutional improvement; on the other hand, by establishing exceptional standards, it may inadvertently reinforce differential expectations for persons with disabilities generally—implicitly suggesting that only those with extraordinary adaptive capabilities can access equal opportunities. More critically, such narratives often overlook the complex strategic negotiation processes behind successful cases and the institutional conditions, social capital, and personal investments that enable these negotiations.

Consequently, this narrative orientation obscures a core issue. When LPwD success requires such “exceptional” personal investment, the critical concern is not differences in individuals’ negotiation capabilities, but rather what characteristics of the institutional environment compel them to mobilize social capital networks and engage in strategic negotiation throughout their legal education. Based on this recognition, this study’s core objective is not to retell inspirational stories, but to treat these publicly documented pioneer experiences as an analytical lens, deconstructing the institutional negotiation strategies and social capital mobilization mechanisms underlying their success to deeply understand the operational characteristics and areas for improvement in China’s legal education and legal professional entry systems.

Through analyzing these pioneer experiences, this study advances the following central argument: The professional development trajectories of pioneering Chinese LPwD should not be understood as simple narratives of individual adaptation, but rather as multidimensional processes of strategic negotiation: in an environment where educational ideals and practical implementation have not fully aligned, they construct foundational support by mobilizing deep bonding social capital and secure professional development opportunities through critical bridging social capital. This process provides an empirical foundation for understanding the alignment between China’s principled commitments to disability rights protection and educational practice in legal education. Importantly, this examination serves not only to understand educational equity within legal training, but also to illuminate how barriers to professional diversity may have downstream implications for health governance capacity—as the perspectives and lived experiences of legal professionals inevitably shape the policies and frameworks that influence population health outcomes.

Based on this argument, this study addresses the following core research questions:

1 Identifying Institutional Environment Characteristics: How do patterns consistent with ableism manifest within standardized legal education design, examination systems, and professional entry mechanisms as challenges for LPwD? How do these challenges reflect the alignment between institutional design and diverse needs?
2 The Dual Role of Social Capital: As formal institutional support systems continue to develop, how does informal social capital perform compensatory functions, and how does this dependence relate to inequalities in opportunity distribution?
3 From Adaptation to Advocacy: How do these pioneers transition from individual “strategic negotiation” to collective institutional improvement actions, leveraging their legal professional identities to promote a paradigm shift from “case-by-case accommodation” to “universal design”?

These three progressively layered questions collectively constitute this study’s analytical framework: from identifying challenges, understanding compensatory mechanisms, and exploring improvement pathways.

To address these questions, this article is structured as follows: First, it constructs and integrates an analytical framework drawing on the analytical lens of ableism, social capital theory, and institutional responsiveness models; second, it presents empirical materials revealing the forms of challenges, functions of social capital, and strategies of agency; third, it deepens understanding of strategic negotiation mechanisms through theoretical dialogue; finally, it proposes policy pathways from individual support to institutional transformation.

## 2 Methodology

Methodologically, this study adopts a secondary qualitative analysis approach, drawing on publicly available narrative texts and official documents to examine how individuals with disabilities experience career development within the legal profession and to identify the underlying social and institutional factors shaping these trajectories. Specifically, this study draws on the methodological framework of document analysis (27), treating publicly available media reports, official website information, and government documents as socially constructed texts—these texts themselves constitute meaningful objects of inquiry, as they not only record individual experiences but also reflect how society understands, presents, and frames the professional trajectories of legal professionals with disabilities (28).

It must be clarified that this study does not treat these public narratives as direct proxies for lived experience, but rather identifies the structural characteristics of the institutional environment, the operational patterns of social capital, and the manifestations of individual agency through narrative texts that have been selected, edited, and framed through media processes. This methodological positioning means that the findings of this study reflect patterns as presented in public narratives, rather than direct representations of individuals’ inner experiences. It should also be noted that the cases included in the analysis are all “success narratives” selected for media coverage, and documents are themselves socially constructed products that have undergone processes of selection and framing (29), meaning that the selection mechanisms of media reporting systematically favor the presentation of positive cases of overcoming barriers. This selectivity means that the data sources of this study contain an inherent survivorship bias: even the successful individuals selected for public presentation encountered formidable institutional difficulties in their journeys, while the challenges faced by those who were not incorporated into public narratives are very likely more severe. Accordingly, the barriers identified in this study should be understood as a lower bound rather than the full picture of the actual barrier spectrum. Furthermore, the goal of this study is not to pursue statistical generalizability, but rather to extract analytical insights through in-depth analysis of representative public narrative cases, in order to understand the alignment between institutional design and diverse needs in China’s legal education and professional entry systems, the compensatory functions and potential limitations of social capital, and the possible pathways through which individuals transition from adaptive adjustment to institutional improvement advocacy.

### 2.1 Theoretical analytical framework

The analytical framework of this study is built upon a core theoretical premise: the degree of inclusivity in legal education may influence the compositional diversity of the legal professional community, which in turn may affect the development of health-related policies and legal frameworks, ultimately connecting to the health equity agenda. This connection from education to health equity remains at the stage of conceptual reasoning at present. To systematically analyze the strategic negotiation of legal professionals with disabilities within institutional barriers, this study constructs an integrative theoretical framework encompassing three dimensions.

First, in identifying institutional challenges, this study took the social model of disability studies as its theoretical foundation and incorporated the concept of “ableism” for deeper analysis. The social model posits that barriers originate not from individual impairments, but rather from the failure of social environments to adequately accommodate the diversity of human functioning (30). The underlying cause of this adaptive insufficiency can be traced to “ableism”—an ideological system that presupposes specific physical and cognitive abilities as normative and systematically privileges this norm in institutional design (31). This study particularly focused on how students with “non-standard” learning and expression methods in elite educational settings such as law schools employ strategic negotiation to adapt to standardized educational design, examination systems, and professional entry mechanisms in response to “academic ableism,” and how these negotiation behaviors reflect the alignment between institutional design and diverse needs.

Second, in understanding individual coping strategies, this study introduced social capital theory as an analytical tool, drawing on its classic distinction between “bonding” and “bridging” capital. Bonding capital emerges within homogeneous groups (such as families and peer communities), providing emotional support and identity affirmation, performing foundational compensatory functions when formal institutional support remains incomplete; bridging capital connects across heterogeneous groups (such as mentors and industry predecessors), providing critical support for individuals’ integration into professional environments and access to development opportunities (32). Simultaneously, this study critically examined the potential “dual effects” of social capital. While helping individuals address challenges, it may also exacerbate inequalities in opportunity distribution, causing persons with disabilities who already lack social capital to face greater difficulties when seeking support.

Finally, in the dimension of evaluating institutional responsiveness models and individual agency, this study established an analytical framework distinguishing between two different support models: “passive reactive” support (such as case-by-case reasonable accommodations) and “proactive preventive” support (such as Universal Design for Learning). The former places greater responsibility for environmental adaptation on individuals, requiring them to apply for and demonstrate need proactively; the latter fully considers diversity from the outset of institutional design, internalizing inclusivity within the system. This distinction not only facilitates assessment of the characteristics of China’s current legal education support system, but more importantly helps understand how pioneers transition from individual strategic negotiation to collective institutional improvement actions, leveraging their legal professional identities to promote a paradigm shift from “case-by-case accommodation” to “universal design.”

These three theoretical dimensions constitute an integrated framework: institutional environment characteristics constitute the field within which individuals must adapt and negotiate; social capital provides critical resources for this process; and the interaction between institutional responsiveness models and individual agency determines the costs and effectiveness of adaptive negotiation.

Furthermore, in connecting these dimensions to health equity frameworks, this study adopts a health governance perspective that views professional diversity within legal systems as a form of institutional capacity relevant to health policy development. This perspective recognizes that legal professionals’ experiential knowledge and perspectives shape their approach to developing health-related legislation, anti-discrimination frameworks, and accessibility standards—thereby linking educational inclusivity to broader questions of governance capacity for health equity (33, 34). This analytical lens enables examination of how barriers in legal education may have implications extending beyond educational equity to encompass the capacity of legal systems to address health-related policy challenges.

### 2.2 Research design and data collection

This study employs a secondary qualitative analysis approach, systematically collecting samples from official media reports, government websites, and other publicly available secondary sources over the past two decades through a purposive sampling strategy, and establishing a textual corpus comprising 14 legal professionals with disabilities after screening. To ensure transparency of case selection and source coverage, sources were identified through systematic searches of Google, China official court and procuratorate websites, the China Disabled Persons’ Federation website, and authoritative Chinese media outlets. Search terms included “legal professionals with disabilities” and “lawyers/judges/prosecutors with disabilities,” covering the period from 2000 to 2024. Texts were included on the basis of three criteria: (1) the subject holds a legal professional qualification or is actively practicing in the legal field; (2) the source originates from official media outlets, government websites, or institutionally authenticated channels; and (3) the text contains sufficient narrative detail to support analysis of institutional barriers and social capital mechanisms. This study adopted a theory-directed analytic approach. Each text was first read in full and annotated for narrative elements relevant to the three framework dimensions: institutional barriers reflecting ableist tendencies, social capital functions and limitations, and individual agency in navigating institutional constraints. In a second stage, annotated passages were compared within and across cases to identify recurring patterns, internal variation, and instances that departed from dominant themes. Interpretations were discussed iteratively among the authors until consensus on pattern characterization was reached. To strengthen analytic credibility, narrative patterns were cross-compared across source types, including media reports, government documents, and official institutional texts, in order to identify consistencies and divergences and reduce reliance on any single source type. The selection of this methodological path over primary empirical data collection was based on the following considerations: at present, systematic data on the career development of legal professionals with disabilities are in a state of absence both in China and globally, with no existing databases or registration systems available for analysis. This study attempted primary data collection through questionnaire surveys in its preliminary phase, but encountered multiple difficulties. First, the population of legal professionals with disabilities is extremely small and highly dispersed, and non-disabled respondents constituted the overwhelming majority of returned questionnaires, resulting in severe selection bias in the data structure and low credibility. Second, the collection of sensitive information involving disability status and professional experiences faces compliance constraints under the framework of the Personal Information Protection Law, which sets strict requirements of legitimacy and necessity for the processing of sensitive personal information, increasing the legal risks of primary data collection. By comparison, publicly available secondary documents possess distinctive methodological advantages: their temporal span covers nearly two decades, enabling the capture of diachronic changes in the institutional environment, and the subjects reported are representative cases with public visibility, providing rich material for analyzing the operational mechanisms of institutional barriers and individual coping strategies.

Furthermore, this study selected the public narratives of these pioneers as analytical objects not to recount their achievements, but to employ them as analytical windows, based on three primary considerations. First, the very “exceptionality” of their success highlights underlying institutional challenges from a particular angle. Second, the patterns of strategic negotiation presented in public narratives demonstrate the critical role played by social capital when formal support systems remain underdeveloped. Third, the trajectories of certain individuals transitioning from personal adaptation to institutional reform advocacy reveal the potential for individual agency to translate into structural improvement. Additionally, the textual corpus exhibits diversity in disability types (visual impairment, hearing impairment, physical disability) and professional distribution (lawyers, judges, procurators, notaries), enabling this study to identify both commonalities and differences in strategic negotiation across different disability types and professional domains.

## 3 Results: navigating the barrier-laden terrain

The textual corpus of this study demonstrates a degree of diversity across multiple dimensions: in terms of disability etiology, it encompasses both congenital and acquired disabilities; in terms of career development trajectories, it includes both direct entry into the legal field and transitions from other domains; in terms of social background, it covers diverse regions, ethnicities, and socioeconomic statuses; in terms of educational background, it spans experiences ranging from special education schools to mainstream university education (Table 1). As discussed in Section 2.2, these cases are all “success narratives” selected for media coverage, and the following findings should be understood in light of this data source characteristic. Based on analysis of this textual corpus, this section systematically presents how the institutional environment creates challenges for legal professionals with disabilities, the functions and limitations of social capital within this context, and the adaptive strategies and agentic practices employed by individuals.

**Table 1:** Research sample: professions, disability types, and primary reported barriers.

No.	Profession	Disability type	Primary barriers	Web site
A	Lawyer	Hearing impairment	Communication barriers, difficulties in legal qualification examination, limited courtroom participation	https://m.news.cctv.com/2021/09/27/ARTIR3jnIIkxbOfakn2qAVn2210927.shtml, https://people.cctv.com/2023/11/05/ARTIezC6fYKKivixmxwzyUwn231105.shtml, https://www.news.cn/photo/2022-07/22/c_1128855694.htm
B	Lawyer	Visual impairment	Information access barriers, screen reader dependence, mobility challenges	http://society.people.com.cn/n1/2024/0129/c1008-40168152.html, https://www.chinanews.com.cn/sh/2023/09-20/10080919.shtml, https://gy.youth.cn/gywz/202401/t20240130_15052159.htm
C	Lawyer	Physical disability	Mobility limitations, physical stamina constraints	https://www.acla.org.cn/info/022722c3de4c40eca4ccec982d8e4dd8, http://www.legalweekly.cn/zfdt/2025-05/26/content_9189210.html, https://www.gzrd.gov.cn/gzdt/dbgz/dbfc/202507/t20250708_88246186.html, https://www.gzrd.gov.cn/gzdt/dbgz/dbfc/202507/t20250717_88298708.html?isMobile=false
D	Lawyer	Physical disability	Wheelchair dependence, restricted mobility, difficulties in daily living activities	https://www.acla.org.cn/info/022722c3de4c40eca4ccec982d8e4dd8, https://www.xn--fiq7vggk68aq1fe49byv8a.xn--zfr164b/n1/2025/0808/c460800-40538690.html
E	Lawyer	Physical disability	Mobility limitations, limited career advancement	https://www.acla.org.cn/info/022722c3de4c40eca4ccec982d8e4dd8, https://www.ynrd.gov.cn/html/2022/daibiaofengcai_1115/19467.html
F	Lawyer/Educator	Physical disability	Daily operational limitations, limited motor abilities, social-psychological barriers	https://www.cdpf.org.cn/hdjl/cjrfc/344f5470b8364a2c9f2b8a03993cb5a6.htm, https://news.gmw.cn/2025-07/01/content_38125722.htm
G	Notary	Visual impairment	Progressive vision loss, work adaptation difficulties, social cognitive bias	https://zqb.cyol.com/pc/content/202507/23/content_414081.html, https://ytdpf.org.cn/xinwenzhongxin/gongzuodongtai/2025-05-29/869.html
H	Prosecutor	Physical disability	Severely restricted mobility, daily living difficulties, work intensity adaptation	https://biaozhang.12371.cn/2012/06/15/ARTI1339762781155999.shtml, https://tv.cctv.com/2013/01/07/VIDE1357563061654114.shtml, https://www.spp.gov.cn/spp/xjym/201907/t20190718_425566.shtml
I	Judge	Physical disability	Writing difficulties, operational inconvenience, professional entry barriers	http://society.people.com.cn/n1/2022/0518/c1008-32424319.html, http://epaper.legaldaily.com.cn/fzrb/content/20220527/Articel03006GN.htm
J	Lawyer	Visual impairment	Textbook access difficulties, insufficient examination time, employment discrimination	https://www.news.cn/local/2022-06/16/c_1128747633.htm, https://www.workercn.cn/c/2022-03-14/6772669.shtml, http://canjiren.china.com.cn/2024-03/29/content_42740298.html
K	Lawyer	Visual impairment	Learning material limitations, examination accommodation needs, insufficient social understanding	https://www.xinhuanet.com/politics/2018-05/20/c_1122859065.htm, https://zqb.cyol.com/html/2020-07/22/nw.D110000zgqnb_20200722_1-07.htm
L	Prosecutor	Physical disability	Prosthetic adaptation difficulties, high physical energy expenditure, heavy economic burden	https://www.spp.gov.cn/ztk/2014/jcz/zmjcg/xgbd/201403/t20140307_68359.shtml, https://www.spp.gov.cn/spp/xjym/201406/t20140609_74145.shtml
M	Law Student	Hearing impairment	Language learning difficulties, intensive rehabilitation training, limited educational resources	http://gxjy.edu.china.com.cn/2024-08/13/content_42893657.htm, http://jyj.liuzhou.gov.cn/xwzx/bmdt/tbbd/t19700101_3507735.shtml
N	Judge	Physical disability	Physical pain and suffering, physical stamina limitations, medical cost burden	https://www.mva.gov.cn/sy/zt/zmtyjrxxhdzl/qttjrx/201811/t20181115_18093.html, https://tv.cctv.com/2022/10/08/VIDELheLxcfS6EQ5EG2UUWKF221008.shtml, http://tv.81.cn/zgjs/lbnh/10190343.html

All cases represent individuals who received media coverage as successful entrants to the legal profession, reflecting an inherent survivorship bias. Barriers listed reflect patterns reported in public narratives and should be understood as a lower-bound estimate of the institutional barrier spectrum rather than a comprehensive account.

### 3.1 The challenge of “standardization”: institutional barriers in legal education and qualification examinations

Analysis reveals that the barriers sample individuals commonly encountered in their development pathways were not isolated individual difficulties but reflected patterns consistent with ableism in institutional design. These institutional barriers compelled students with disabilities to engage in high-cost strategic negotiation during their legal education and qualification examination processes, as they navigated pressures to “prove” their qualifications within mainstream educational systems.

Several individuals faced non-linear early educational trajectories as a direct consequence of disability onset, including multi-year interruptions, redirection to vocationally-oriented special education schools, and placement in mainstream environments without adequate support infrastructure. Individual A, for instance, experienced a five-year educational interruption following acquired hearing loss, while Individual B persisted in mainstream high school after a visual impairment diagnosis despite the absence of institutional support. Across these cases, a consistent pattern emerges: when mainstream systems lacked the capacity to accommodate non-standard learning needs, the burden of adaptation fell on individuals rather than institutions. Those who remained in mainstream settings consistently improvised personal compensatory strategies in ways that reflect systemic gaps in environmental design rather than individual deficiency.

These cases demonstrate that when corresponding support systems remained incomplete, LPwD’s educational pathways frequently confronted a difficult choice: whether to obtain specialized support within a special education system emphasizing vocational education, or to pursue development through extraordinary personal effort within standardized mainstream environments. This situation exhibited a pattern consistent with “academic ableism”: when educational systems center on a singular “normal” standard, responsibility for environmental adaptation is primarily transferred to individuals (35).

As the core gateway to the legal profession, the National Unified Legal Professional Qualification Examination intensively exemplified the challenges brought by this “standardization.” Through its standardized examination format, it equated “legal competence” with the ability to complete standardized tests within time limits (36). Individual B’s experience constitutes a critical case revealing the dual dilemma disabled candidates face: he had to perform a risk assessment between “concealing disability to avoid discrimination” and “disclosing disability to obtain support.” He initially chose to conceal his disability status during the initial application stage precisely because institutionalized support channels remained unclear, preventing him from anticipating the consequences of disclosure. His subsequent proactive disclosure of disability to seek accessibility support after passing the initial screening was a form of strategic negotiation—first ensuring entry into the system, then seeking adaptive adjustments. The local judicial bureau’s positive response, establishing a separate examination room for him, was lauded by the media as successfully implementing the “reasonable accommodation” principle in practice.

However, a deeper analysis of this case reveals its contingency. The acquisition of accommodations was not a proceduralized, stable right; instead, its outcome depended heavily on three non-institutional factors: the individual’s effective advocacy strategy, the enlightened attitude of specific personnel handling the case, and the local institution’s resource allocation capacity. This precisely reflected the inherent limitations of the “passive reactive” support model—the realization of rights depends on contingent factors in case-by-case processing rather than stable, reliable institutional guarantees.

Furthermore, accessing accessible learning resources exposed deeper support deficiencies. For example, Individual A with hearing impairment struggled to find teaching videos equipped with subtitles, meaning she had to invest several times more time and energy than others to understand the duplicate content. Individual J with visual impairment obtained learning materials through crowdsourced collaboration with volunteers. Hundreds of volunteers from across the country photographed and scanned paper versions of legal examination textbooks page by page, then identified, proofread, and transcribed them into digital versions, producing over a dozen electronic textbooks totaling over 2 million characters.

The patterns of barriers in legal education and qualification processes revealed in public narratives, if representative of broader institutional practices, may have implications extending beyond individual career trajectories. When institutional environments systematically filter professional entry based on factors other than legal competence, the resulting professional communities may lack the diversity of perspectives necessary for developing truly inclusive health-related policies and anti-discrimination frameworks, and this conceptual connection warrants future empirical investigation through direct examination of health policy development processes.

### 3.2 Family and community as compensation: the dual effects of bonding social capital

Analysis of public narrative texts reveals a recurring theme: in the context of underdeveloped formal institutional support, “bonding social capital” constituted by informal networks such as families and peer communities performs a critically important compensatory function in individual development. However, it should be noted that public media narratives may systematically emphasize the “touching” aspects of family support while underrepresenting cases of failure due to insufficient family resources, meaning that the familial compensatory functions presented in this analysis may contain a positive bias.

Families, particularly parents, constituted the first and most stable support system in individuals’ development pathways (37). Across cases, family support operated across multiple life stages and took varied forms: sustaining educational persistence in the face of external pressure to withdraw, enabling transitions between professional domains, and providing the basic daily assistance that made continued participation in demanding professional environments physically possible. Individual H’s situation illustrates the foundational nature of this dependence: the severity of his physical impairment meant that basic daily functioning required ongoing family assistance, making family presence a structural precondition for professional participation rather than merely supplementary encouragement. This sustained cross-stage support demonstrated the family’s stabilizing function during multiple identity transitions.

However, the necessity of such family support reflected room for improvement in the institutional environment. When formal social capital—such as educational systems—failed to provide inclusive support, families made extraordinary sacrifices to fill institutional gaps. Family “resource devotion” encompassed not only financial investment, but also sacrifices of time, energy, and even career development—this total family mobilization support model highly privatized responsibilities that should have been borne by public education. While this close family support network embodied the power of bonding social capital, the very necessity of its “safe harbor” function reflected an issue requiring attention: in a genuinely inclusive institutional environment, individuals should not need to rely so heavily on families to realize fundamental educational rights (38). More critically, this dependence on bonding social capital formed a hidden filtering mechanism: persons with disabilities possessing sufficient family capital (economic, cultural, and emotional) were more likely to obtain development opportunities, while those with scarce family resources might face disproportionate difficulties and challenges from the outset.

Peers and communities, meanwhile, constructed mutual aid networks based on shared experiences. The formation of these networks did not arise from choice, but rather from LPwD’s proactive strategies in seeking belonging and solutions within mainstream professional environments. For instance, Individuals B and H’s career trajectories were closely connected to disability community development. Similarly, the “Xiao Ai Bang Bang” (Little AI Helper) public welfare platform for blind people initiated by Individual K provided remote assistance to persons with visual impairments through volunteer video connections, cumulatively providing legal services to over 600 persons with disabilities. They not only served this community but also drew strength from it, forming a collaborative network based on shared identity and experience through joint development of assistive technologies, establishment of public welfare platforms, and organization of professional training (39). This network provided not only concrete instrumental support but, more importantly, reinforced group identity and collective efficacy.

However, it should also be noted that while this bonding based on homogeneous identity fulfilled important functions of bonding social capital, overreliance on or overemphasis of homogeneous communities might limit LPwD’s interaction and integration with the mainstream legal profession while obtaining internal support, potentially imperceptibly delaying systemic influence on mainstream institutions.

The reliance on informal social capital networks represented in public narratives, while enabling some individuals to succeed, raises questions about representativeness in legal professional communities. If professional success depends heavily on family resources and fortuitous connections rather than institutionalized support, the resulting professional community may systematically underrepresent perspectives from specific socioeconomic backgrounds. At a conceptual level, this filtering effect may limit the range of experiential knowledge available in health policy development, and this connection warrants future empirical investigation through direct examination of health policy formulation processes.

### 3.3 Critical linkages in career development: opportunities and contingencies of bridging social capital

If bonding social capital primarily addressed foundational issues of survival and identity, then “bridging social capital”—connections with heterogeneous groups such as mentors, industry predecessors, and key institutions—this form of informal social capital provided critical opportunities for individuals to transcend environmental constraints and achieve professional advancement.

Across cases, bridging capital operated through three recurring channels: mentorship from established practitioners, coordinated intervention by sympathetic institutional actors, and proactive outreach from professional organizations. What these channels share is their contingent character, as each depended on timing, personal visibility, or the disposition of specific individuals rather than on stable, universally accessible institutional pathways. Individual A’s apprenticeship under a nationally recognized sign language lawyer provided entry into a specialized professional network that would otherwise have remained inaccessible. Individual I’s employment in the court system was secured only through coordinated intervention by the provincial disabled persons’ federation after standard physical examination requirements had blocked his entry despite outstanding examination results. Individual J was proactively recruited by a law firm following his qualification examination success, an invitation that enabled a career transition from informal work to formal legal practice. In each case, the opportunity was real and consequential, but its emergence was unpredictable and non-reproducible by design.

However, analysis of these cases indicates that the acquisition of bridging social capital often possessed strong contingency and unpredictability. Whether Individual A’s encounter with Tang Shuai or Individual I’s coordinated with key departments, the emergence of these opportunities was highly dependent on non-institutionalized factors such as specific timing, interpersonal networks, and personal characteristics. This dependence transformed professional success into a “game of luck” rather than rights protection. More critically, this dependence on bridging social capital likely further exacerbated inequalities in opportunity distribution: those individuals who could access key mentors, obtain support from institutional actors, or be discovered by professional institutions often already possessed a certain degree of social visibility, personal charisma, or prior achievements—this itself constituted a form of filtering. Therefore, from an institutional development perspective, the future direction should be to establish more universal and stable support systems, reducing individuals’ dependence on contingent opportunities.

At a conceptual level, the significance of these bridging capital connections may extend beyond individual career development. Mentors and institutional actors who facilitate the entry of persons with disabilities into professional fields may also serve as critical channels for integrating diverse perspectives into legal professional communities, perspectives that may subsequently inform more inclusive health-related policy development. However, engaging in these social exchanges entails implicit constraints: the reciprocity mechanisms embedded in social ties may limit recipients’ autonomous action space (40), and the expectations of capital providers can shape recipients’ career pathway choices, potentially restricting individual freedom (41). This suggests that legal professionals with disabilities who entered the profession through such social capital may be constrained to some extent in their directional choices in activities such as public health legal decision-making and policy formulation. It should be noted that the data in this study capture the operational processes of these bridging mechanisms but do not directly trace the actual impact of these professionals on health policy—this pathway from professional entry to policy outputs warrants verification through future research.

### 3.4 Continuous adaptation in professional practice: dual labor and adaptive accommodation

Notably, according to the Lawyers Law of the People’s Republic of China, graduating from law school and passing the qualification examination does not mark the endpoint of legal education. Graduates must complete a one-year internship at a law firm before they can apply for formal practice. Therefore, the initial stage of professional practice remains institutionally an extension of legal education. In this critical transition from student to professional, certain constraints that align with ableism became more implicit. The challenges individuals with disabilities faced shifted from relatively explicit entry barriers to more subtle and persistent social attitude challenges, compelling them to engage in dual labor: The first layer consisted of the high-intensity professional labor required by legal work itself (42); the second layer was the additional adaptive labor necessary to maintain a “normal” appearance within an environment reflecting ableist tendencies labor whose core purpose was not enhancing professional competence, but rather concealing or compensating for one’s “non-standard” characteristics (22).

This adaptive labor was first manifested in communication and work methods. For example, Lawyer A had to learn pronunciation from scratch to communicate with hearing colleagues and judges, adapting to a work environment centered on oral communication. This unidirectional adaptation requirement reflected the system’s limitations in accommodating diverse communication modalities. Second, it manifested in the continuous management of technology and the environment. Individual B always wore headphones while working to prevent his screen reader from disturbing the shared office space. Individual G’s work pattern was even more typical: he constantly wore headphones, with his screen reader set to 3.0x playback speed to improve efficiency, striving to keep pace with other colleagues’ work efficiency. This consciousness of “not causing trouble for others” reflected individuals’ compromises to integrate into shared work environments.

Deeper adaptation involved responding to attitudinal barriers from within and outside the profession. Individual A faced challenges such as “How can you speak of justice when you cannot even appear in court?” Individual H encountered similar questioning: “Who has ever seen a prosecutor appear in court in a wheelchair or on crutches?” This insistence on “standard” professional image led him to choose prosthetics over a wheelchair. Such challenges and self-adjustments reflected stereotypical perceptions of images of legal professional roles. They required LPwD to demonstrate professional competence and invest extra effort in proving their conformity to the legal professional image. These adjustments constituted continuous, invisible adaptive labor.

This adaptive labor requiring constant proof of capability was typically invisible, unpaid, and rarely formally recognized. However, it continuously depleted individuals’ cognitive and emotional resources, constituting the hidden costs they faced long-term in professional fields. Its root cause lay in the disproportionate placement of responsibility for environmental adaptation upon individuals, while optimization of the environment lagged relatively behind.

The patterns of “dual labor” represented in public narratives, if prevalent in broader professional practice, may have implications extending beyond individual career trajectories. The continuous depletion of cognitive and emotional resources is not merely a personal cost but may, at a conceptual level, constitute an additional institutional barrier that limits these professionals’ capacity to engage in the “transformative agency” discussed later, such as participating in pro bono work or policy advocacy related to health equity. Consequently, the “hidden costs” of navigating a professional environment marked by ableist tendencies may inadvertently limit the sustained engagement of professionals who possess the very perspectives most needed to inform inclusive health policy development, and this connection from professional exhaustion to constrained policy participation warrants future verification through direct examination of professionals’ actual policy engagement.

## 4 Discussion: from individual adaptation to systemic transformation

The patterns revealed in the preceding analysis point to three interrelated dynamics that together illuminate the institutional challenges facing legal professionals with disabilities and the conceptual connections to health governance capacity. The discussion begins with the structural role of social capital: when informal social capital is elevated from a “beneficial supplement” to a de facto prerequisite for professional success, opportunity distribution ceases to hinge on professional competence itself and becomes deeply tied to individuals’ capital endowments, producing a filtering effect that poses a potential threat to the representational diversity of legal professional communities. Building on this, the discussion traces the institutional roots of this predicament—the prevailing “passive reactive” accommodation model places disproportionate responsibility for adaptation on individuals, while a paradigm shift toward “proactive preventive” Universal Design offers a reform pathway that shares a common logic with upstream intervention strategies in public health. Finally, the discussion shifts from institutional constraints to the actors themselves, examining how pioneers who successfully entered the legal profession have translated their individual adaptive experiences into institutional advocacy. This trajectory from passive coping to active transformation may be understood, within health governance frameworks, as an emergent form of empowered social participation.

### 4.1 The centrality of social capital and the reproduction of opportunity inequality

As systematically revealed by the case analysis in Section 3, one of the core findings of this study is that, within the current institutional environment, the importance of informal social capital for LPwD’s professional success has transcended the category of “beneficial supplement” to become a practically indispensable prerequisite. This indicates that in an institutional environment that has not fully overcome the influence of ableism, individual career achievement depends not only on professional competence or personal effort, but to a large extent on whether one is fortunate enough to possess sufficient informal social capital (43, 44). Bonding capital (such as unconditional family support) and bridging capital (such as mentorship from key guides) together constitute the critical pathways through which they traverse institutional barriers.

The deeper implication of this phenomenon is that overdependence on informal social capital may imperceptibly reproduce or even deepen existing social inequality structures. When individual success is highly dependent on the capital endowment of one’s family of origin and fortuitous encounters with key “guides,” a “dual exclusion mechanism” may form: on one hand, institutional environments with ableist tendencies create challenges for the universal participation of persons with disabilities (45); on the other hand, uneven distribution of social capital further filters out those persons with disabilities lacking corresponding resources (46). A “Matthew effect” in legal education thus emerges: opportunity distribution tends to favor individuals who already possess social capital advantages, potentially creating greater obstacles for persons with disabilities from lower socioeconomic status families when entering the elite legal profession (42, 47). As this effect accumulates across generations, disability representation within the legal professional community will increasingly exhibit elitization trends, and this class homogeneity will precisely erode its representational foundation for the entire disability community.

Functionally, this pattern objectively constitutes a responsibility transfer: support functions that should be provided by formal institutions (such as education and employment systems) have been transferred to the private sphere, particularly families. This privatization of public responsibility, while demonstrating through a few pioneer success stories the possibility of individuals overcoming difficulties, may also diminish the urgency of improving institutions themselves. These “success stories” can easily be interpreted as evidence that “existing institutions are fully viable,” potentially obscuring the experiences of the larger majority who failed to succeed due to lack of similar support.

Therefore, this study’s findings point to a fundamental institutional issue: the true inclusive transformation of China’s legal education should not aim to teach more persons with disabilities how to engage in high-cost strategic negotiation, but rather should strive to reduce or even eliminate the institutional barriers that make strategic negotiation necessary; its pathway should not be to expect more “exceptions” to emerge, but rather to make inclusivity a universal environmental norm obtainable through normal effort alone.

From a public health perspective, the patterns revealed in this section suggest potential connections to health equity frameworks. The filtering effect created by unequal access to social capital shares certain logical similarities with mechanisms that stratify access to health resources and opportunities (48, 49). Research on social determinants of health has consistently demonstrated that socioeconomic status—itself often shaped by educational opportunity structures—constitutes a fundamental cause of health disparities (50). Although this study focuses on legal education rather than health outcomes directly, the findings point at a conceptual level to a governance concern warranting attention: when legal professional communities involved in developing health-related policies and anti-discrimination frameworks systematically underrepresent persons with lived experience of disability, the resulting governance structures may lack the critical perspectives necessary to formulate truly inclusive health policies (51, 52).

### 4.2 From passive response to proactive prevention: prospects for universal Design in China’s legal education

The cases in this study clearly demonstrate that China’s legal education and related professional entry systems currently adopt a “passive reactive” case-by-case processing model when addressing disability issues. Understanding this distinction is important not only for educational reform but also for recognizing how institutional design choices may influence the governance capacity of legal systems to address health equity. This model’s characteristic lies in maintaining the centrality of a “standard” pathway while treating individuals’ diverse needs as exceptional circumstances requiring “special handling.” (53). Whether B’s examination accommodations obtained through personal appeal or I’s employment opportunity secured through multi-party coordination, both exemplify this model’s operational logic: the system presupposes a singular, standardized pathway; when individuals not conforming to this standard appear, a post-hoc, remedial procedure is initiated. Under this model, the responsibility for articulating needs, demonstrating needs, and validating legitimacy rests primarily with individuals. At the same time, the stability and predictability of outcomes depend largely on individuals’ negotiation capabilities and institutions’ goodwill.

A more forward-looking institutional paradigm is “proactive preventive” Universal Design for Learning (UDL). The core of UDL lies in treating diversity as normative rather than exceptional from the outset; it no longer adheres to the binary framework of “normal” versus “special,” (54) but rather acknowledges that all learners possess their own uniqueness (55). Therefore, it advocates pre-building flexibility and multiple options into all aspects—including curriculum design, teaching methods, material presentation, and student assessment—making the educational process inherently more accessible to everyone, including students with disabilities (56). For example: providing subtitles for course materials by default (beneficial not only for students with hearing impairments, but also enhancing all students’ learning convenience in noisy environments); ensuring textbook compatibility with assistive technologies (because technological enhancement is everyone’s right); offering diverse assessment methods (such as project reports and oral presentations, acknowledging the multiple manifestations of legal competence) rather than relying on singular timed closed-book examinations and post-hoc accommodation requests (57).

Promoting China’s legal education paradigm shift from “passive response” to “proactive prevention” is not merely a technical adjustment, but a profound educational philosophy revolution. When inclusivity is embedded in environmental design, individuals no longer need to engage in additional “negotiation” to obtain basic learning support. Also, reasonable accommodations transform from post-hoc special arrangements into default attributes inherent in educational environments. In this regard, the “anticipatory duty” principle in the UK’s Equality Act may offer valuable reference—requiring institutions to proactively assess and remove potential barriers rather than passively awaiting individual complaints (58). Notably, promoting such improvements (such as pedagogical innovations) can largely be achieved by Chinese universities through their institutional autonomy (59), and does not necessarily depend on high-cost macro-policy changes or legal amendments.

**Figure 1:**
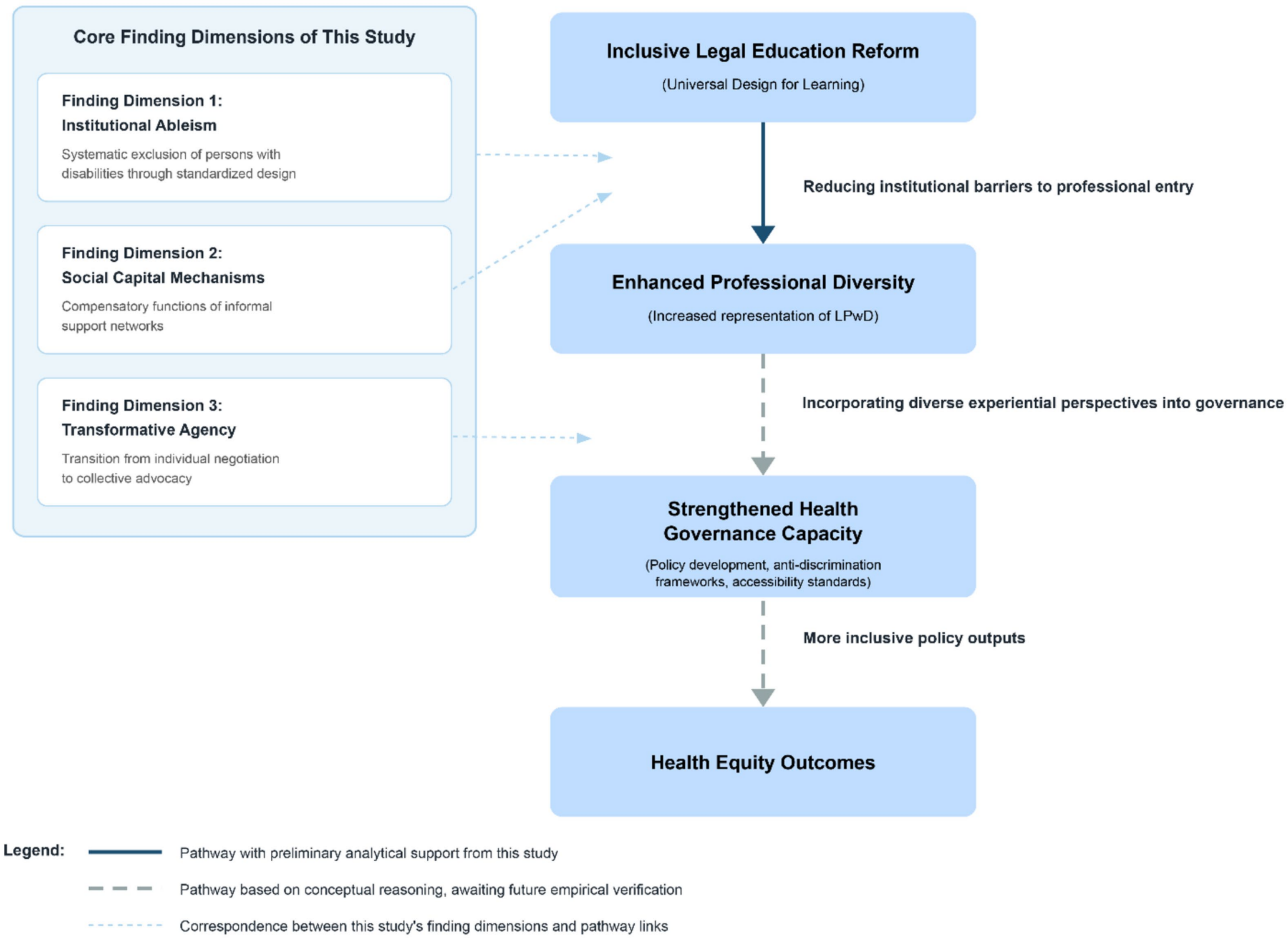
Hypothesized conceptual pathway: from inclusive legal education to health equity.

The paradigm shift from “passive reactive” to “proactive preventive” approaches discussed in this section resonates with broader frameworks in public health intervention design. Universal Design principles align conceptually with upstream prevention strategies that address structural determinants of health rather than treating individual cases *post hoc* (60, 61). In the health equity context, upstream interventions aim to modify the fundamental conditions that generate disparities, rather than merely responding to disparities after they emerge (62, 63). We propose the following conceptual analogy: when applied to legal education, the principle of proactive inclusivity—by reducing barriers to professional entry and development—may enhance the capacity of legal systems to function as effective governance mechanisms for health equity. Conceptually, legal professionals who bring diverse experiential perspectives may be better positioned to identify gaps in health-related legislation, advocate for accessibility standards in healthcare settings, and ensure that anti-discrimination frameworks adequately address the intersection of disability and healthcare access, though this proposed pathway requires direct empirical verification in future research (64, 65). Accordingly, promoting Universal Design in legal education may be conceptualized as a form of capacity building for health governance. Figure 1 presents this conceptual pathway from inclusive educational reform to health equity outcomes and identifies the current level of evidential support at each link.

### 4.3 The transformative role of professional agency: from individual experience to institutional improvement

Another important finding of this study is that the pioneers in the sample did not merely passively adapt to or overcome barriers, but demonstrated high levels of agency, dedicating themselves to leveraging their professional identities to participate in and promote institutional improvement. Their choice to enter the legal field itself possessed strategic significance. Law is not merely a profession; it is one of society’s most central institutional tools (66), a normative discourse system concerning rights, equality, and justice (67). By becoming legal professionals, they acquired the institutional power and professional legitimacy to deploy this discourse, enabling them to elevate personal adaptive experiences into systematic reflection on and advocacy for institutional reform. This transformation from individual adaptation to institutional advocacy represents a form of empowered participation particularly relevant to health governance—as legal professionals are uniquely positioned to influence the frameworks that shape health-related policies and protections.

This transformation signifies an epistemological shift: from “how do I adapt to this standardized system” to “why is this system designed this way,” and further to “how do we improve this system.” Drawing on their professional identities, they were able to enact a critical “narrative shift”: redirecting public attention from “inspirational” stories focused on individuals overcoming physical impairments to examining the institutional and environmental factors creating social barriers. When Lawyer B participated in public interest litigation promoting barrier-free transportation, he was reshaping individual needs into universal rights issues through public judicial channels. When Lawyer A committed herself to ensuring deaf people “are no longer outsiders to the law” and dedicated herself to promoting deaf persons’ equal participation in the judicial system, she was advancing the inclusive progress of the entire legal system.

Their practice demonstrates that legal professionals are not merely implementers of legal rules, but can also become active agents promoting the self-improvement of legal systems. Their experiences reveal pathways toward deeper social progress: the key may lie not in teaching more persons with disabilities strategic negotiation skills, but in creating an institutional environment requiring no special “negotiation”; the emphasis may lie not in cultivating more “exceptional individuals,” but in continuously examining and reforming the universal rules that give rise to “exceptional status.”

The transformative agency demonstrated by the legal professionals in this study offers insights relevant to empowerment frameworks within public health discourse. Empowerment, as conceptualized in health equity literature, involves not only individual capacity development but also the ability to participate meaningfully in governance structures that shape health-related policies (68, 69). When persons with disabilities successfully enter legal professions and subsequently advocate for institutional reform, they exemplify a form of empowered social participation that extends beyond individual adaptive success to encompass structural advocacy (70, 71). However, this study’s data can only present the trajectories of some individuals from experiencing institutional barriers to undertaking institutional advocacy, and these narratives sketch a possible pathway from capacity building to empowerment to governance participation. Accordingly, at a conceptual level, inclusive legal education may serve not only as an equity issue in itself, but also as a potential strategic intervention for building the governance capacity necessary to advance health equity more broadly, a proposition that awaits empirical substantiation.

## 5 Research limitations and future directions

This study has several limitations. First, it primarily relies on publicly available secondary sources, meaning the sample is concentrated on “successful” cases that have garnered media attention. As discussed in Sections 2.2 and 3, this selectivity produces a survivorship bias, and the experiences of persons with disabilities who encountered greater resistance within the system, failed to complete their studies, or were unable to enter the legal profession remain systematically absent from this analysis. Their “invisibility” itself constitutes an issue urgently requiring in-depth research. Second, the connection to health equity is presented primarily as a conceptual framework (Figure 1), and the pathway from inclusive legal education to professional diversity, to health governance capacity, and ultimately to health equity outcomes involves assumptions at each link that require independent verification. Third, this study employs a secondary qualitative analysis methodology, with conclusions aimed at achieving analytical generalization rather than statistical inference. The findings are most applicable to understanding institutional dynamics within the Chinese context; generalization to other countries or legal systems should be approached with caution.

To address these limitations and deepen related research, future scholarly exploration could proceed in three directions. First, employing primary data collection methods such as in-depth interviews and ethnography to more broadly engage persons with disabilities with diverse experiences (including those who succeeded, withdrew midway, or failed to enter the profession), thereby obtaining richer, more multidimensional narratives—their experiences of “failure” may more profoundly reveal the true character of the institutional environment. Second, building upon qualitative research, larger-sample quantitative studies could test this study’s hypotheses regarding the correlations among social capital, institutional support, and professional achievement. Third, future research should directly examine the actual participation and influence of legal professionals with disabilities in health-related policy-making to empirically test the conceptual pathway proposed in this article from inclusive legal education to health governance capacity.

## 6 Conclusion

Through secondary qualitative analysis of public narratives concerning 14 Chinese LPwD, the patterns identified in this study suggest deep institutional issues with implications that may extend beyond educational equity to encompass health governance capacity. The public narratives examined in this study suggest that the emergence of these pioneers should not be understood as simple triumphs of individual resilience, but rather as outcomes of individuals pursuing adaptive accommodation through high-cost strategic negotiation in an institutional environment that has not fully overcome the influence of ableism. The necessity of such negotiation reflects, from one perspective, areas where the institutional environment requires improvement: in a more ideal legal education system, students with disabilities’ realization of equal educational and developmental rights should rely primarily on stable, universal institutional guarantees rather than extraordinary personal effort and fortuitous social capital.

Patterns identified in these narratives point to a three-dimensional interactive mechanism: First, institutional environments with ableist tendencies appear to not only create challenges for individuals, but also tend to place responsibility for adaptation primarily on individuals; second, while informal social capital performs compensatory functions, it may also obscure the need for institutional optimization and correlate with unequal opportunity distribution, causing the scales of success to tilt more easily toward individuals possessing abundant social capital; third, individual agency demonstrates the transformative potential from passive adaptation to active advocacy, providing possibilities for promoting incremental institutional improvement. This mechanism raises concerns about an inherent tension in current legal talent cultivation for persons with disabilities. In practice, the system’s formal openness appears to rely considerably on individuals’ extraordinary efforts and informal resources for its maintenance.

These findings raise questions with potential implications for both educational equity and health governance. When institutional barriers limit professional diversity within legal systems, the resulting governance structures may lack the range of perspectives necessary for developing health-related policies that adequately address diverse population needs. Addressing these barriers may thus represent a dual investment—in educational inclusivity and in the governance capacity necessary for advancing health equity.

Based on these findings, to promote more substantive inclusive development in China’s legal education regarding disability issues, this study offers reflections at three levels for related policy and practice:

1 Discursive Reorientation: From Celebrating Individuals to Building Systems. The focus of policy discourse and public communication could gradually shift from highlighting a few “exemplary” individuals toward constructing universal, predictable support systems. While affirming individual agency, greater emphasis should be placed on the long-term value of institutional development to prevent “exceptionism narratives” from obscuring more universal institutional issues.
2 Paradigmatic Innovation in Institutional Models: From Passive Response to Proactive Design. It is recommended that China’s law schools and legal professional qualification certification institutions actively explore transitioning from the “reasonable accommodation” passive response model toward the “Universal Design for Learning” (UDL) proactive prevention model. This means integrating inclusive thinking into the entire process of curriculum design, teaching methods, assessment approaches, and physical environment construction, making it a default attribute of institutions rather than post-hoc special arrangements.
3 Structural Optimization of Support Channels: From Dependence on Informal Networks to Strengthening Formal Guarantees. Reducing individuals’ overdependence on informal social capital lies fundamentally in establishing institutionalized support systems. This includes but is not limited to: establishing well-resourced, accessible learning support centers within law schools, conducting systematic training for faculty and staff, establishing transparent and efficient accommodation application and response procedures, and providing stable funding guarantees. The goal is to enable every student with disabilities to access development opportunities through stable, reliable channels rather than relying primarily on “fortunate” personal circumstances.

Ultimately, this study advances a conceptual argument: promoting inclusivity in legal education may be conceptualized as an upstream intervention in the broader health equity agenda. By addressing institutional barriers that limit professional diversity, legal education reform may enhance the capacity of governance institutions to develop health-related policies and legal frameworks that more adequately address the needs of marginalized populations. When legal systems that shape health-related policies reflect greater professional diversity, they may be better equipped to identify gaps in health protection frameworks, advocate for accessibility standards in healthcare settings, and ensure that anti-discrimination legislation adequately addresses the intersection of disability and health access. Through a secondary qualitative analysis of public narratives concerning Chinese legal professionals with disabilities, this study articulates the theoretical plausibility of the conceptual pathway from inclusive legal education to health governance capacity, rather than providing empirical evidence that such a pathway currently exists (Figure 1), suggesting how the complex interplay among capacity building, social participation, and empowerment may constitute structural factors influencing health governance. These insights provide a preliminary conceptual foundation for future empirical research that directly examines the relationship between legal professional diversity and health policy quality.

## Data Availability

The original contributions presented in the study are included in the article/supplementary material, further inquiries can be directed to the corresponding author.
